# TREMSUCS-TCGA – an integrated workflow for the identification of biomarkers for treatment success

**DOI:** 10.1515/jib-2024-0031

**Published:** 2024-12-11

**Authors:** Gabor Balogh, Natasha Jorge, Célia Dupain, Maud Kamal, Nicolas Servant, Christophe Le Tourneau, Peter F. Stadler, Stephan H. Bernhart

**Affiliations:** Interdisciplinary Center of Bioinformatics, 9180 Leipzig University, Härtelstraße 16-18, D-04107 Leipzig, Germany; Bioinformatics Group, 9180 Institute of Computer Science, Leipzig University, Härtelstraße 16-18, D-04107 Leipzig, Germany; Department of Drug Development and Innovation (D3i), Institut Curie, Paris, France; Inserm U900 Research Unit, Saint Cloud, France; Université Paris-Saclay, 91190 Gif-sur-Yvette, France; Max-Planck-Institute for Mathematics in Sciences, Inselstraße 22, D-04109 Leipzig, Germany; Leipzig Research Center for Civilization Diseases (LIFE), University Leipzig, Härtelstrasse 16-18, D-04107 Leipzig, Germany; Department of Theoretical Chemistry of the University of Vienna, Währingerstrasse 17, A-1090 Vienna, Austria; Center for RNA in Technology and Health (RTH), Univ. Copenhagen, Grønnegårdsvej 3, Frederiksberg C, Denmark; Facultad de Ciencias, Universidad Nacional de Colombia, Bogotá CO-111321, Colombia; Santa Fe Institute, 1399 Hyde Park Road, Santa Fe NM 87501, USA

**Keywords:** TCGA, SCC, biomarker, precision medicine, differential expression, differentially methylated regions

## Abstract

Many publicly available databases provide disease related data, that makes it possible to link genomic data to medical and meta-data. The cancer genome atlas (TCGA), for example, compiles tens of thousand of datasets covering a wide array of cancer types. Here we introduce an interactive and highly automatized TCGA-based workflow that links and analyses epigenomic and transcriptomic data with treatment and survival data in order to identify possible biomarkers that indicate treatment success. TREMSUCS-TCGA is flexible with respect to type of cancer and treatment and provides standard methods for differential expression analysis or DMR detection. Furthermore, it makes it possible to examine several cancer types together in a pan-cancer type approach. Parallelisation and reproducibility of all steps is ensured with the workflowmanagement system Snakemake. TREMSUCS-TCGA produces a comprehensive single report file which holds all relevant results in descriptive and tabular form that can be explored in an interactive manner. As a showcase application we describe a comprehensive analysis of the available data for the combination of patients with squamous cell carcinomas of head and neck, cervix and lung treated with cisplatin, carboplatin and the combination of carboplatin and paclitaxel. The best ranked biomarker candidates are discussed in the light of the existing literature, indicating plausible causal relationships to the relevant cancer entities.

## 1 Introduction

### 1.1 Scope of this study

A plethora of public data repositories provide access to a rapidly increasing body of biomedical research data. Among these are large databases containing genomic, transcriptomic and epigenetic data [1, 2] linking these to medical data [3]. The sheer volume of data, however, makes the resourced difficult to use without a sound expertise in big data analysis. In order to make efficient use of the stored information in practise requires elaborate task-specific workflows and interfaces that support high-level queries posed by experts in biomedicine. One of these tasks is the discovery of potential transcriptomic or epigenetic markers for treatment success.

In order to address the problem, we developed TREMSUCS-TCGA (see List of abbreviations). The software implements a workflow to query the data from TCGA. The treatment(s) to be analyzed as well as the cancer types can be chosen freely by the user. While being flexible to the users request, the pipeline is completely automated. Built upon the workflow management system Snakemake [4], TREMSUCS-TCGA guarantees reproducibility. Optional parameters facilitate a modulation of the TCGA given survival data or influence postprocessing survival-analysis based ranking of potential biomarkers. The pipeline can be started with a single terminal command. An interactive mode assists the selection of cancer types and the respective therapeutic regimes. A single final html report file aggregates all relevant results. It holds descriptive statistics from both, the patient meta data included and the differential analysis results. Also Kaplan Meier plots from invoked survival analyses are processed and well arranged. If feasible, commonalities of found potential biomarkers between the different created sets are aggregated as well. The github repository is hosted at [5] where an example report file can be downloaded and the whole documentation is also linked.

This contribution is organized as follows: First, we will give a short overview of the TCGA-project as it is used as data source for the medical and biomolecular data for this study. Already established tools based on TCGA are briefly introduced as well. Second, we will define the problem at hand in more detail and describe our workflow. We then apply our software to a showcase example concerning treatment alternatives for squamous cell carcinomas.

### 1.2 The TCGA project

The Cancer Genome Atlas (TCGA) [6], a cancer genomics program, sequenced and molecularly characterized over 20,000 primary cancer samples and spans 33 cancer types. It is led by the National Cancer Institute‘s (NCI) [7] Center for Cancer Genomics (CCG) [8] and the National Human Genome Research Institute (NHGRI) [9]. The joint effort of TCGA began in 2006, bringing together researchers from diverse disciplines and multiple institutions. The data collected by this initiative can be accessed via the gdc dataportal [1], which is hosted at the GDC [10]. A comprehensive list of publications by TCGA is given at [11]. This list is constantly updated as the TCGA Analysis Network continues to study and mine the data.

Results created within the scope of the project are either made directly available through the program’s researchers or through independent researchers who utilized the data. Raw data (e.g. BAMs), germline and non-validated mutations, are under controlled access. Derived data, however, is available in open access. Research associated with the TCGA project already lead to improvements on the ability to diagnose, treat and prevent cancer [12, 13]. In addition, many tools, web-applications and workflows have been created and established to help researchers with their ongoing analysis. A collection of some tools which have been developed to analyze TCGA data can be found at [14].

These tools provide a wide spectrum of descriptive and analytic functionalities also for data-types not invoked in our study here. The Cancer Imaging Archive TCIA [15], for example, provides access to radiological imaging data sets in DICOM format from TCGA cases. It supports phenotype-genotype research for cancer imaging analysis. FASMIC [16] is an integrative and analytic web platform for annotating functional impacts of somatic mutations in human cancer and TANRIC [17, 18] is an integrative web server for analyzing lncRNAs in cancer.

Tools and platforms that correlate patients survival data with biomarkers also have become available. With help of user provided inputs, a biological network file, a molecular profiling file and a patient survival data file, SurvNet searches for network-based biomarkers [19]. Preloaded datasets can also be invoked for further inspections. Existing applications, however, are typically webservices that lack some cornerstones properties that would ensure reproducibility, which is a key requirement in modern data analysis [20].

## 2 Methodology of the pipeline

### 2.1 Overview of the workflow

**Figure 1:**
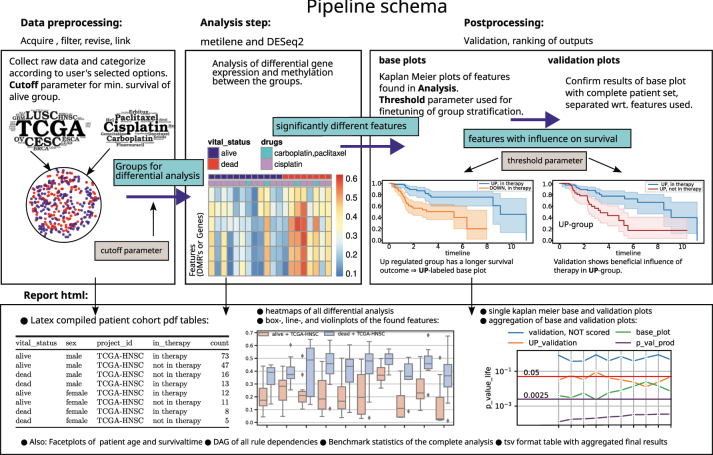
Schema of the TREMSUCS-TCGA pipeline. Top: Schema showing workflow. Results passed between steps have cyan background. Bottom shows examples of results in the report.

TREMSUCS-TCGA analyzes survival data for subsets of the TCGA project focussed on a user-supplied list of cancer types and treatments. The workflow of TREMSUCS-TCGA, can be subdivided into three main steps: preprocessing, differential analysis, and post processing, as shown in Figure 1. In preprocessing, the patients are categorized into groups representing positive (alive per default) or negative (dead per default) treatment outcomes. In the Analysis step, these groups are then investigated to find differential features (either differentially expressed genes or differentially methylated regions) between them. In postprocessing, these features are first analysed wrt. their impact on survival, defining marker candidates. The last step of postprocessing is analysing possible markers to find the ones that are treatment specific.

**Table 1:** Filtering compositions of the survival function classifications.

Base plot	UP validation	DOWN validation
UP	UP	UP|DOWN
DOWN	UP|DOWN	DOWN

The repository is hosted at Github [5]. A detailed description for the usage and the installation via conda can be found at the documentations page at [21] and the example report can be downloaded at [22] or directly viewed within a browser at [23].

During the preprocessing steps, data is acquired from the GDC-TCGA database [1]. A complete list of available cancers is given in Table A1. Data filtering and downloads are directly implemented on the basis of the GDC database release version 31 [24]. Raw count data or methylation data is downloaded for the user’s choice of cancer types and therapeutic combinations. To ensure that patients are grouped correctly and to avoid information loss due to spelling errors or inconsistencies in drug names, a revision of the drug names given within the linked metadata is performed. A complete list of corrections performed can be seen in Table A2. After the linking of the meta-data to the given raw-data, the methylome and trancriptome analyzing parts are set up accordingly and the input for the subsequent analysis steps is prepared.

During the analysis steps, the patient data is grouped according to the respective vital status. For transcriptomic analysis, we employ DESeq2 [25], a tool for differential gene expression analysis based on the negative binomial distribution. For methylation data, we invoke metilene [26] which uses a binary segmentation algorithm combined with a two-dimensional statistical test for DMR detection.

After these analyses, survival estimations are performed for the most significant differential expressed genes or DMRs found by comparison of the vital status groups. The genes or DMRs included into subsequent analyses must surpass an adjusted *p*-value of 0.05 (DESeq2) or a *q*-value of 0.05 (metilene). Moreover, the number of genes included is limited to a maximum of 60 for upregulated and 60 for downregulated genes respectively. The same limitation holds for DMRs. The set of input data is divided into high (up) and low (down) expression/methylation groups using the mean of medians of the vital states as pivot element (see Section 2.3).

This classification is then used for the first Kaplan Meier plots of the validation step, which we refer to as the *base plots* (see Figure 1, postprocessing). They include the set with the selected drug combinations. The significance of the differences in the KM plot is estimated through the *p*-value of a Cox proportional hazards (PH) fitter. Both, the Kaplan Meier and the Cox analyses make use of the lifelines library [27] for python.

Assessment of the possible usefulness of a biomarker for treatment success requires a comparison with data from patients that did not receive the treatment under consideration. A valid treatment-related biomarker is significant for the respective treatment, but has a different characteristic for other treatments. Otherwise, the biomarker is not treatment specific, but universal, and has no effective significance for choice of treatment. Accordingly, the patient cohort is regrouped and data points without treatment with the applied therapeutic combination are included in the subsequent survival analysis.

For both the UP and DOWN groups, a Kaplan Meier estimation is performed between the treated or not treated patients. We refer to these plots as the UP and DOWN validation plots, respectively. To illustrate this, if the base plot suggests a better treatment outcome for patients with an up regulated marker (**UP** labeled), then the UP validation estimation should show a higher life expectancy for patients in therapy (then also **UP** labeled, see Table 1 and the example illustration within Figure 1 of the postprocessing step.) Correspondingly, if the base plot shows a better life expectancy with a down regulated marker, the patients in treatment must have a better life expectancy in the DOWN validation. For simplicity, the schema in Figure 1 just shows the situation for an UP labeled base plot, where the UP validation confirms that patients in therapy have a longer survival expectation. Note that the progression of the “UP, in therapy” group between the base plot and the UP validation plot differs slightly, because the classification method (see Section 2.3) is applied on different patient cohorts due to the inclusion of the complete patient set into the validation plots. The grouping of the patients and the validation steps can be refined using the cutoff and threshold parameters (see Sections 2.4 and 2.5).

Based on all traits gathered, a comprehensive aggregation and ranking of the putative markers for treatment success is subsequently performed. To this end, the significance values obtained from the base and the corresponding pair of validation plots are combined (see Section 2.6 for details).

The results are collated in a final html page (example report at [22]) that presents all relevant outcomes in tabular and graphical form. In addition, comparative analyses depending on automatically created patient cohorts (see Section 2.2) are included as well. The visualization includes heatmaps derived from the differential analyses, the Kaplan Meier plots created in the survival analysis as well as scatter and lineplots aggregating intermediate or final results. The report makes use of built-in features Snakemake such as the creation of benchmark statistics, the visualization of the job dependencies as a DAG and the logging of the exact versions of tools and packages involved. The source codes of the particular script file is also associated with the respective jobs performed.

In the remainder of this section the individual components of the workflow are described in detail.

### 2.2 Creation of datasets for analysis

In a prospective clinical study, the design and composition of the patient cohort must be well considered beforehand [28] s.t. the cohorts are consistent and just differ in the condition that is to be differentially analysed, for example the vital status of dead and alive. In a retrospective approach such as TREMSUCS-TCGA, this complete cohort harmonisation is not possible anymore. Hence, it is a good practice to regroup the available cases according to potential confounding factors like cancer-type or sex and to integrate a comparative analysis of the biomarkers found across these groups. The creation and combination of datasets that are analysed with the differential methods (i.e., DESeq2 or metilene) is therefore part of TREMSUCS-TCGA. Some sets might contain too few cases to do meaningful analysis s.t. the aggregated sets across those factors are also considered for the comparative analysis.

**Figure 2:**
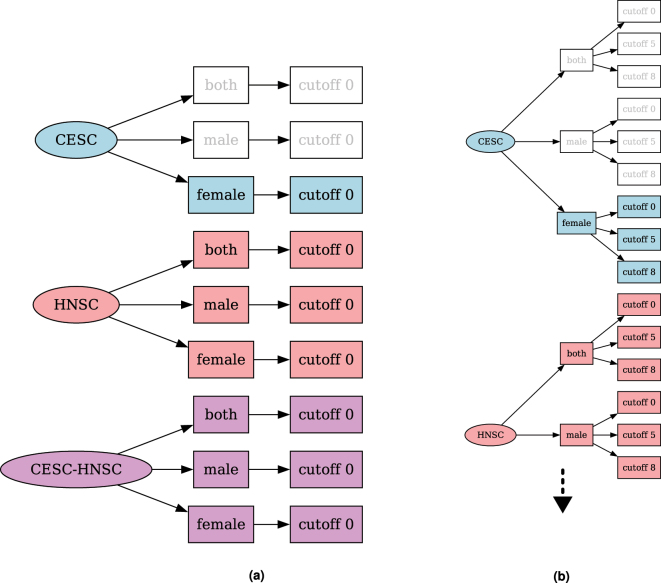
Combinatorial trees for a TREMSUCS-TCGA run with the cancers TCGA-CESC and TCGA-HNSC. (a) Combinatorial tree of sets which would be differentially analysed for a TREMSUCS-TCGA run with the cancers TCGA-CESC and TCGA-HNSC and the default cutoff. (b) Combinatorial tree of sets which would be differentially analysed for a TREMSUCS-TCGA run with the cancers TCGA-CESC and TCGA-HNSC with custom cutoffs of 5 and 8.

**Figure A1:**
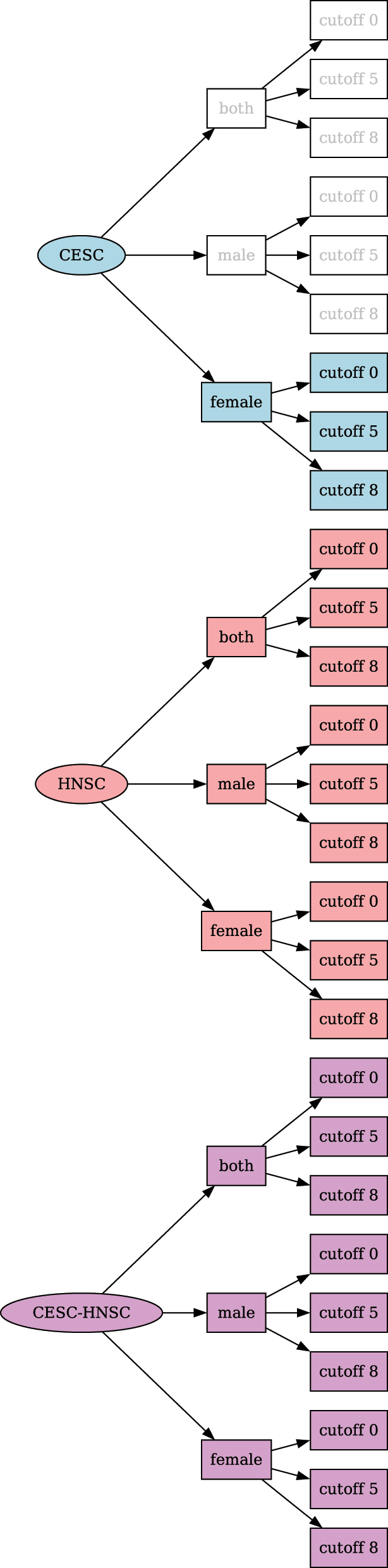
Complete combination tree of the cohorts created for a TREMSUCS-TCGA run with the cancers TCGA-CESC, TCGA-HNSC and additional custom cutoff parameters of 5 and 8.

For each cancer type used in a TREMSUCS-TCGA run, three distinct patient sets are analyzed, one for each sex and one for the combination of both. If more than one cancer type is analyzed, an additional basket trial of pooled data in the three sex sets is performed, as can be seen in Figure 2a which shows the setup for an example run with the two cancers TCGA-CESC and TCGA-HNSC. For TCGA-CESC, no male cases are available and thus only the females set is created and subsequently analyzed. For each custom cutoff parameter (see Section 2.4), an additional new set per cancer type/sex combination is analyzed. This is illustrated in Figure 2b, where in addition to the default cutoff of 0, the custom cutoffs of 5 and 8 are applied to the TREMSUCS-TCGA run. Thus, the number of differential analyses rises from 7 in Figure 2a to 21 in Figure 2b (truncated for space limitation, fully shown within Figure A1).

By default, both DESeq2 and metilene analysis are performed for all generated sets. However, the user can restrict analysis to one of the two analysis types. If both analyses are selected, an aggregation of all results over both analysis types is integrated in the final Snakemake html report.

### 2.3 Pivot element for UP and DOWN classification

**Figure 3:**
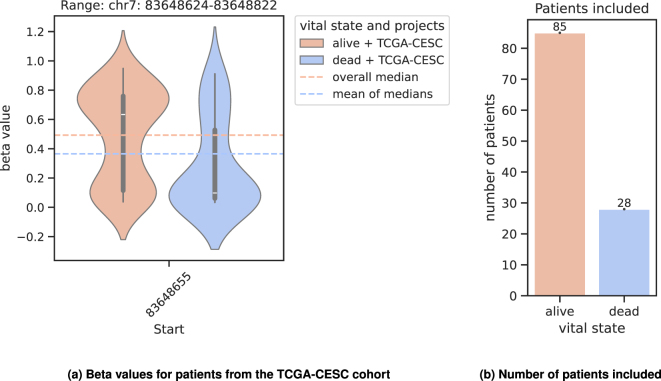
Determination of the pivot element. (a) Example set for patients out of the TCGA-CESC cohort, treated with cisplatin, carboplatin and the combination of carboplatin and paclitaxel. Both, the cutoff and threshold parameter (see Sections 2.4 and 2.5) are set to their default value of 0. The distribution of the beta values at position 83,648,655 out of the DMR *chr*7: 83,648,624 − 83,648,822 is shown. The overall median is calculated with *median*(*alive* + *dead*), the mean of medians (*mom*) is calculated with *mean*(*median*(*alive*) + *median*(*dead*)). The position belongs to *ENSG*00000170381 with the gene name *SEMA*3*E*. (b) Number of patients included into the analysis on the left.

The binary patient survival metadata for the variable “vital status” from the TCGA is given by the values of “dead” and “alive” and will also be referred to as such in the following. To avoid statistical imbalances stemming from an uneven distribution of dead and alive patients within the data sets, we use the mean of medians as pivot element distinguishing between UP and DOWN regulation (Figure 3). Both metilene and DESeq2 already address unbalanced group sizes in their respective estimation procedures, hence no further processing is necessary. Metilene invokes a two-dimensional Kolmogorov–Smirnov test (2D-KS) [29], which is very stable as a non-parametric test. DESeq2 uses techniques such as empirical Bayes shrinkage for fold-change estimation, the Wald test, or optionally a likelihood ratio test (LRT), which helps to maintain robustness even with unequal group sizes.

In the example in Figure 3, 85 alive cases and 28 dead cases are included. It is clearly visible that the median of the complete set (overall median, Figure 3a) is shifted towards the median of the alive cases (white rectangle), suggesting an unbalanced pivot element. In contrast to that, the mean of medians does not show this imbalance and thus gives a more reliable separator.

### 2.4 The cutoff parameter

**Figure 4:**
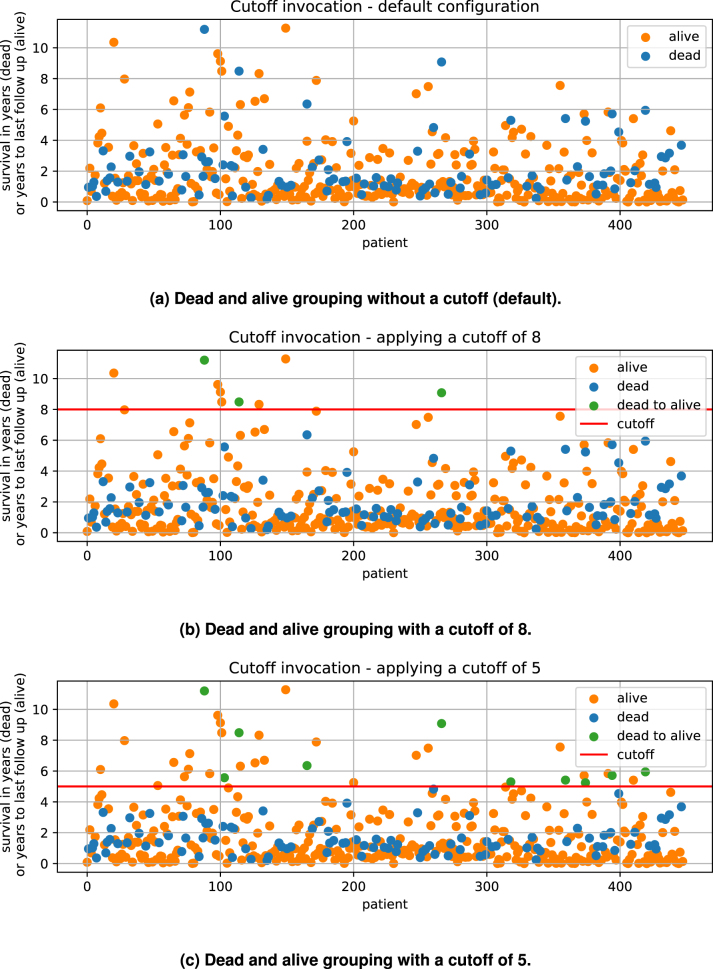
Cutoff example. In (a) the given default survival status of all cases available out of TCGA-CESC, TCGA-LUSC and TCGA-HNSC without any limitation to the treatment is shown. For dead cases, the survival entry is given with the variable “survival in years” and for alive cases it is given with “years to last followup”. (b) Shows the modification applied with a cutoff of 8 years and (c) with a cutoff of 5 years.

It may be desirable form a medical point of view to re-classify what is regarded as a positive outcome of the treatment. Accordingly, the cutoff parameter can be used to replace the “vital status” classification with a classification based on a minimum survival time. If the parameter is set, patients are assigned to a group depending on whether or not they survived longer then the specified value. In Figure 4 an example is given for patients out of CESC, HNSC and LUSC without any limitation to treatment. With a cutoff of 8 years, 3 dead patients are grouped with the alive cohort (Figure 4b). Applying a cutoff of 5 groups an additional 7 dead cases to the alive cohort (Figure 4c). As indicated in Figure 1, this parameter is applied before the analysis steps. In Section 3.5 the impact of the cutoff parameter on the example set and the predicted biomarkers is discussed. The modification of the survival data of just a few patients can have a noticeable impact on the overall outcomes. We advise changing these values based on medical expertise. If a data based cutoff value has to be chosen, the user can perform a run with the default cutoff. The survival data of the given cohort is shown in the “patient_overview” section of the report thus created. This can then be used to determine a reasonable cutoff value. Note that a second run with a new cutoff will lead to the same result as if both, the default and the custom cutoff would have been started together, since the default is always calculated and incorporated within the analysis and earlier results are not overwritten. It is also possible to apply multiple custom cutoff values to one run, see Figure 2.

**Figure 5:**
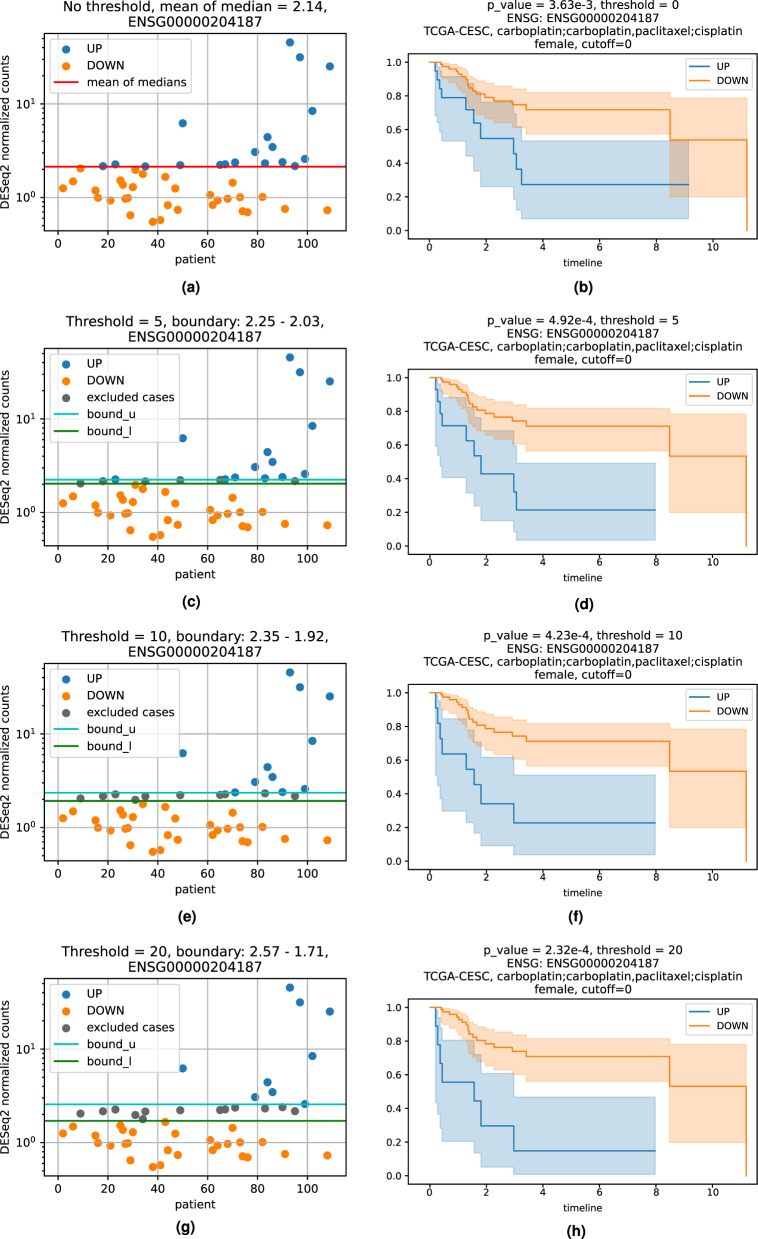
Threshold example for ENSG00000204187. The panels on the left side show the exclusion of patients which are linked to the data in between the threshold bounds. On the right side the belonging Kaplan Meier plot is shown. (a) Inclusion of all cases without threshold. (b) Survivalplot with all cases. (c) Exclusion of cases with threshold of 5. (d) Survivalplot, exclusion of cases with threshold of 5. (e) Exclusion of cases with threshold of 10. (f) Survivalplot, exclusion of cases with threshold of 10. (g) Exclusion of cases with threshold of 20. (h) Survivalplot, exclusion of cases with threshold of 20.

### 2.5 The threshold parameter

The threshold parameter facilitates a modulation in the validation steps. Each previously identified marker, either a differentially methylated position or a differentially expressed gene of each patient, is grouped into the UP or DOWN regulated set depending on the mean of medians of all values. In the following, the Kaplan Meier estimations for each of these two groups are calculated. Any stratification based on a single value can lead to a situation where two or more data points with very similar values are put into different groups. Thus, incorporating values close to the mean of medians might be detrimental to the significance of the survival analyses. With the threshold, an upper (*bound*_*u*) and lower (*bound*_*l*) bound (see Figure 5) around the mean of medians is calculated and patient-data between those boundaries is excluded from the survival analysis. Here, the threshold gives the distance of the bounds from the mean of medians in percent of the mean of medians.

**Figure 6:**
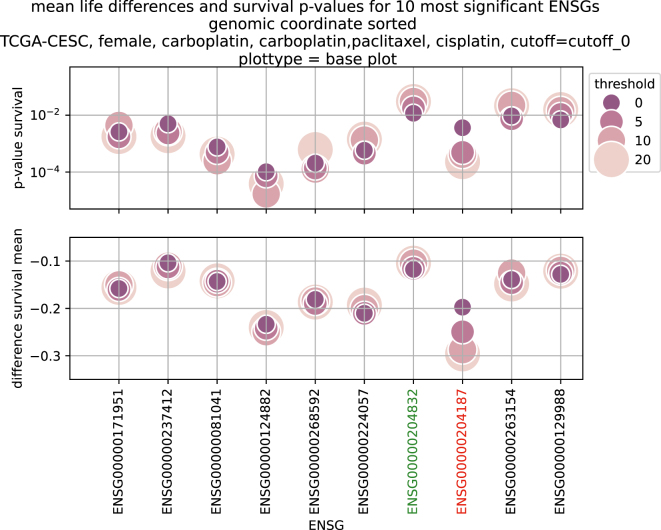
Survival *p*-values and mean life differences for the first 10 most significant genes found by DESeq2, gathered from base plots (see Figure 1), with a cutoff of 0. Succession of ENSGs is genomic coordinate wise.

In Figure 6, the survival *p*-values of the 10 most significant genes for patients from the TCGA-CESC cohort with the therapeutic combination of carboplatin, carboplatin and paclitaxel (combined) and cisplatin are shown. With increasing threshold, incrementally improvement of the *p*-value for ENSG00000204187 (emphasized in red) is visible together with a higher difference of the life expectancies. Increasing the threshold will lower the size of the data base for *p*-value estimation, which can also result in increasing *p*-values. In Figure 6, an example is the gene ENSG00000204832 emphasized in green. Both genes are used here to showcase potentially monotonic changes in dependence of varying thresholds. Apart from that, their relevance in cancer research has already been confirmed through several puplications. ENSG00000204187 encodes for C10orf136 which is also known as LINC00619, a lincRNA which is linked to gastric cancer, osteosarcoma and Hirschsprung’s disease (30], [31], [32). ENSG00000204832 encodes for ST8SIA6-AS1, which belongs to the lncRNAs and is found to be associated with liver cancer ([33, 34]).

For the final ranking and filtering of biomarker candidates, the lowest *p*-value per gene is incorporated into the subsequent steps described in Section 2.6.

For the methylation analysis, the selection for the threshold dependent *p*-value has to be performed slightly differently because DMRs per definition contain multiple positions with different beta values.

**Figure 7:**
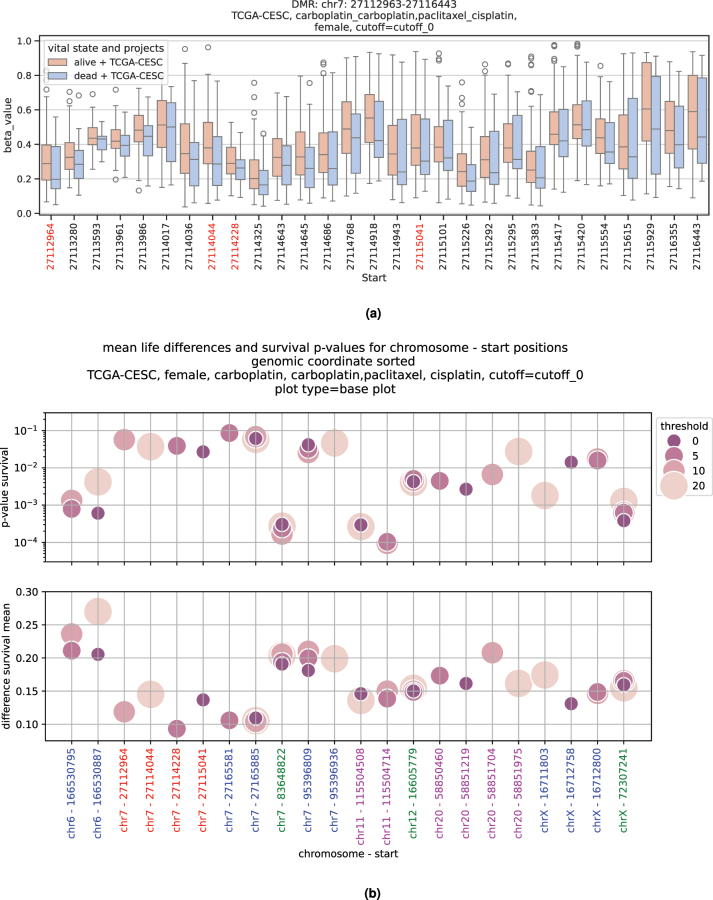
Aggregated metilene survival analysis results for female patients out of the TCGA-CESC cohort, treated with carboplatin, carboplatin and paclitaxel (combined) and cisplatin. (a) Beta values for all positions of the DMR from chromosome 7 between 27,112,963 and 27,116,443. (b) Chromosome – start based outcomes for metilene.

For every DMR position a Cox regression test is performed with each of the threshold values. The positions where a given threshold performs best are recorded. For each DMR thus multiple alternative positions may be obtained as shown in Figure 7a (selected positions are highlighted in red). Figure 7b displays the corresponding survival *p*-values and the mean survival differences of the positions gathered from all DMRs found with the female patient set of TCGA-CESC treated with carboplatin, cisplatin and carboplatin together with paclitaxel. The positions estimated through the DMR from the upper plot 7a are again highlighted in red. The alternating blue and purple lettering in this plot distinguishes positions belonging to different DMRs. Green labels indicate that all 4 threshold values perform best on the same position of the respective DMR.

Similar to the genes found with the DESeq2 approach, here, the best survival *p*-value per position is incorporated into the subsequent steps for the final ranking and filtering described in Section 2.6. We do not advise to exceed a threshold value of 20, since the number of excluded patients makes it unlikely to gain significance for the survival analysis. As for the cutoff parameter, in addition to customized thresholds applied by the user, the default value of 0 is also calculated.

### 2.6 Combination and ranking of the validation plot pairs

**Figure 8:**
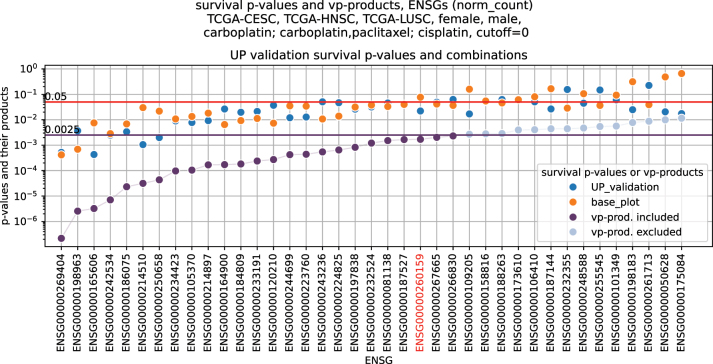
Illustration shows the ordering of the vp-products. 25 genes have a vp-product below the limit and are included in the final result. The example set consists of TCGA-CESC, -HNSC, and -LUSC for male and female patients, cutoff used is 0. The gene marked red corresponds to a case where a low *p*-value in the validation offsets the not significant *p*-value in the base plot.

For the final result, biomarker candidates are ranked based on the product of the *p*-values (henceforth vp-product) of the corresponding base and validation plots. If the vp-product is below a limit of 0.0025, we consider a biomarker a valid candidate. If the threshold parameter was used in a TREMSUCS-TCGA run, the minimal (i.e. optimal) vp-product for every biomarker candidate is used (Figure 8). In this procedure, a low *p*-value in one of the two combined plots can offset a *p*-value above 0.05 in the other, so that the vp-product is still below the limit. An example is ENSG00000260159 in Figure 8.

## 3 Results

### 3.1 A showcase example

In the following, results obtained with the combination of the squamous cell carcinomas of TCGA-CESC, TCGA-HNSC and TCGA-LUSC, treated with cisplatin, carboplatin and the combination of carboplatin and paclitaxel, are shown. Furthermore, cutoffs of 0 (default), 5 and 8 are applied as well as threshold values of 0 (default), 5, 10 and 20. The terminal command issued was:

TREMSUCS -p TCGA-CESC -p TCGA-HNSC -p TCGA-LUSC -d cisplatin

-d carboplatin, paclitaxel -d carboplatin -o OUTPUT_PATH

−*t* 5 −*t* 10 −*t* 20 −*C* 5 −*C* 8 −c 40

**Figure 9:**
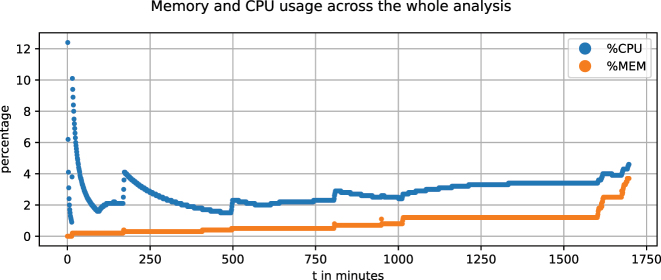
Gradient plot of relative memory consumption and CPU usage on a system with 40 cores and 125 GB of ram.

The −*c* 40 option here causes the underlying Snakemake tool to run in parallel on 40 cores. This triggers 37,502 completed jobs in total, which took about 1 day, 4 h and 24 min corresponding to an estimated 17 days, 7 h and 23 min on a single core. We logged memory and CPU usage on our environment with 40 cores and 125 GB of RAM, taking a snapshot every minute over the course of the whole analysis. Figure 9 show that the memory consumption is not exceeding 3.7 % (4.7 GB). The supplemental material given within the github repository [35] holds the underlying data. Apart from the memory and CPU benchmarks, the run and creation times are part of the statistic section in the final report [22] and all results discussed in the following can be found there as well.

#### 3.1.1 Patient composition of the example set

In Table 2, the composition of our example data set w.r.t. vital status, sex and cancer type is shown. As some data can be missing for some patients, the total number of cases is different for transcriptome (448) and methylome (434) analysis. Patients not treated with the selected drug combination are exclusively used for the validation steps.

**Table 2:** Patient numbers for TCGA-CESC, TCGA-HNSC, TCGA-LUSC, for transcriptome and methylome analysis. A total of 448 cases are included in the transcriptome, and 434 in the methylome analysis.

			In therapy		Not in therapy	
Vital status	Sex	Cancer	# transcriptome	# methylome	# transcriptome	# methylome
Alive	Female	TCGA-CESC	83	85	17	17
Alive	Male	TCGA-HNSC	64	72	43	47
Alive	Female	TCGA-HNSC	11	12	10	11
Alive	Male	TCGA-LUSC	10	9	69	57
Alive	Female	TCGA-LUSC	6	5	25	18
Dead	Female	TCGA-CESC	28	28	10	10
Dead	Male	TCGA-HNSC	14	14	16	16
Dead	Female	TCGA-HNSC	7	8	5	5
Dead	Male	TCGA-LUSC	2	1	23	14
Dead	Female	TCGA-LUSC	0	0	5	5
Total			225	234	223	200

**Figure 10:**
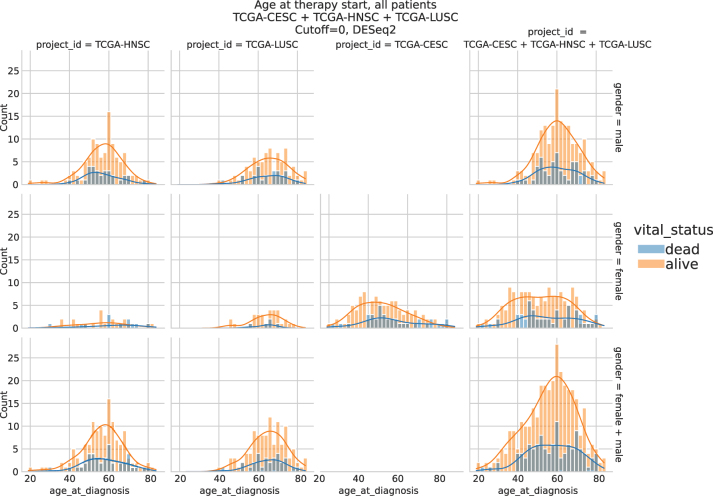
Facet plot of the patients age included into the example set analyses. No limitation to treatment.

In Figure 10, the age distributions of the patient sets created for the analysis are shown. As TCGA-CESC has female cases only, 10 different analysis are performed.

Based on the user’s choice of cancers and drug combinations, tabular decompositions (see Table 2 for an example) and facet plots such as Figure 10 of the patient sets are produced as part of the final Snakemake report.

#### 3.1.2 Analysis specific result comparison

**Figure 11:**
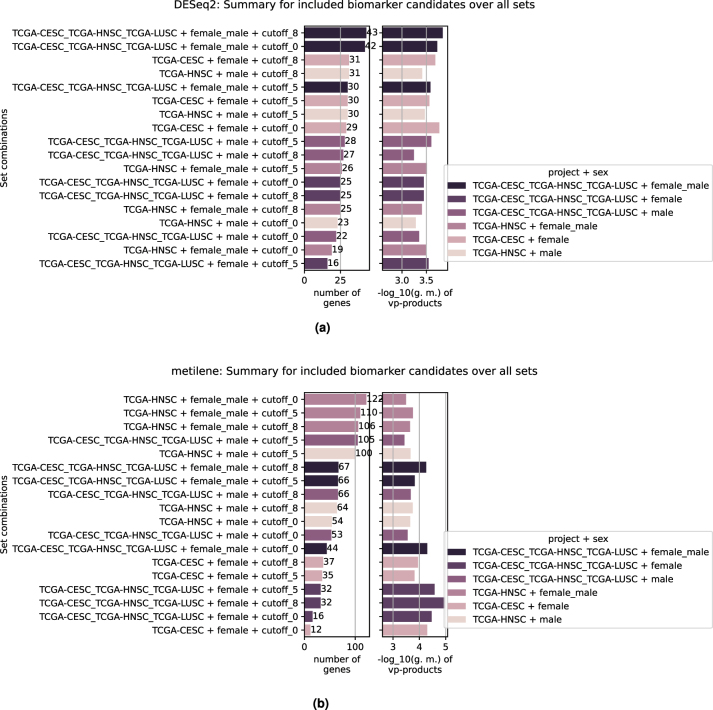
All biomarker candidates found that passed the combined *p*-value validation (Section 2.6). (a) Number of biomarker candidates derived from the DESeq2 analyses. (b) Number of biomarker candidates derived from the metilene analyses.

For both transcriptome and methylome analysis, a compilation of the potential biomarkers is produced. All biomarkers that passed the filtering steps (see Section 2.6) are represented in these summary representations, see Figure 11.

In Figure 11a, the aggregated and ranked results for the transcriptome analysis of the sample-set is shown. It contains, for every set combination where candidates were found, the geometric mean of the validation products and the respective number of invoked genes. The color indicates the same patient cohort. The results are ordered by the number of genes. Figure 11b shows the aggregation of the methylome analyses. All aggregated results which are collected within both plots are accessible via the final Snakemake report and their underlying data can be downloaded directly from the report for further detailed inspection. Beside of features shown here, the intermediate results are presented as figures and tables, where values such as log2fc, *p*-vlaue, adjusted *p*-value or number of CpGs are shown if applicable.

### 3.2 Biomarker candidates from the example run

In this section we briefly discuss the biomarker candidates, i.e., the genes listed in Tables 3 and 4. These are the 5 top ranked genes in terms of the vp-products (see Section 2.6) for transcriptome and methylome analysis, respectively. Within recent literature, most of those genes are already reported to be either predictive, prognostic or at least diagnostic biomarkers, albeit not necessarily in the three squamous cell cancer types analysed in our example.

**Table 3:** Top five biomarker candidates found in the transcriptome, sorted by the validation products. Regulation relative to the group with longer survival.

Cancer	Sex	Cutoff	Gene name	Regulation	Validation products	log2FoldChange
CESC-HNSC-LUSC	Female-male	Cutoff-8	SPIB	UP	3.91*e* − 08	−2.604771
CESC	Female	Cutoff-0	EREG	DOWN	5.02*e* − 07	2.598291
HNSC	Female-male	Cutoff-0	FADD	DOWN	6.79*e* − 07	1.201673
HNSC	Male	Cutoff-5	RP11-20B7.1	DOWN	8.46*e* − 07	2.665860
CESC-HNSC-LUSC	Female-male	Cutoff-8	RORB	UP	9.10*e* − 07	−2.925838

**Table 4:** Ordered after the validation products. Five best biomarker candidates found on the basis of DESeq2. Regulation refers to the group with longer survival expectation.

Cancer	Sex	Cutoff	Gene name	Regulation	Validation products	mmd
CESC-HNSC-LUSC	Female	Cutoff-8	SALL1	UP	2.51*e* − 09	0.063997
CESC-HNSC-LUSC	Female-male	Cutoff-0	MSX2	UP	5.38*e* − 09	0.064413
CESC-HNSC-LUSC	Female-male	Cutoff-8	TRAPPC9	UP	1.31*e* − 08	0.164241
CESC-HNSC-LUSC	Female-male	Cutoff-8	FLRT2	UP	1.98*e* − 08	0.077226
CESC-HNSC-LUSC	Female-male	Cutoff-8	RP11-497E19.1	UP	1.98*e* − 08	0.077226

### 3.3 Potential predictive biomarkers from the transcriptome

The strongest indication for a predictive biomarker based on the expression data was found with SPIB (Spi-B Transcription Factor). SPIB belongs to the Ets family of transcription factors which is known to be involved in tumor progression [36]. It is involved in B cell receptor-mediated signal-transduction [37] and its prognostic value and immunological role were revealed in a comprehensive pan-cancer analysis [38].

The second candidate, EREG (epiregulin), is a risk factor for the prognosis and a potential target for drug development of patients with cervical cancer [39]. It is a member of the epidermal growth factor family and plays a vital role in the progression of the cancer by triggering the EGFR (epidermal growth factor receptor) signaling pathway. In breast cancer, EREG is part of a multi-gene co-regulation system together with lnc021545 and miR-330-3p that affects the metastasis of breast-cancer [40]. The cooperation of these three factors may provide a more accurate marker for anti-metastasis therapeutic and prognostic evaluation. EREG is also involved in the progression of head and neck squamous cell carcinomas [41], where its loss has been suggested as a predictive biomarker for patients that might benefit from a combination of ferroptosis inducers and cetuximab.

FADD (Fas-associated protein with death domain) was initially identified as an adaptor protein for death receptor-mediated extrinsic apoptosis [42]. Its dysregulation has been shown to be closely associated with the pathogenesis of numerous types of cancer 43], [44], [45. A recent review [46] summarized the role of FADD in cancer progression and discusses clinical implications as a biomarker and therapeutic target for cancer patients.

The candidate RP11-20B7.1 ENSG00000242741 is a long non-coding RNA corresponding HGNC gene symbol LINC02005. No functional information is available for this gene.

Finally, RORB (Retinoid-Related Orphan Receptor Beta) is one of the circadian clock genes. It was found to be associated with immune evasion and is suggested to serve as a potential marker of cancer [47].

### 3.4 Potential biomarkers from the methylome

For the metilene-based outcomes, SALL1 (Spalt Like Transcription Factor 1) was found to give the strongest indication for a predictive biomarker out of the female cohort with the combination of all 3 cancers and a cutoff of 8, see Table 4. SALL1 promoter hypermethylation was found to correlate with reduced disease-free survival in patients with head and neck squamous-cell carcinoma [48] and is suggested as an important biomarker.

The second putative marker is MSX2 (Msh Homeobox 2) methylation. MSX2 has been implicated to have a role in breast and pancreatic cancer [49]. It is found to correlate significantly with increased recurrence-free survival and is concluded to may be an important regulator of cell invasion and survival. It could also be shown that an accumulation of MSX2 leads to a sensitization of breast cancer cells to tamoxifen [50].

As a regulator of the cytokine-induced NF-*κ*B signaling pathway, which has been proven to play pivotal roles in the progression of various malignancies [51], TRAPPC9 (Trafficking Protein Particle Complex Subunit 9) over-expression could be shown to be closely related to tumor differentiation, depth of invasion, clinical stage and lymphatic metastasis in gastric cancer. Also a TRAPPC9 dependent, NF-*κ*B signalling pathway mediated Lapatinib resistance could be revealed in Lapatinib-resistant ERBB2-amplified breast cancer cell lines [52].

FLRT2 (Fibronectin Leucine Rich Transmembrane Protein 2) was identified as a tumor suppressor in a global study on epigenetic regulations and biological functions [53]. Epigenetic silencing of FLRT2 contributed to the pathogenesis of CRC. Moreover, FLRT2 promoter methylation may be a useful epigenetic biomarker for the prevention and treatment of CRC.

RP11-497E19.1 was found among six novel risk signatures that can effectively assess prognosis and provide potential therapeutic targets for STAD (gastric adenocarcinoma) patients by Peipei Xu et al. [54]. In this study, novel lncRNA gene signatures were developed by identifying angiogenesis-related genes to better predict prognosis in STAD patients.

### 3.5 Impact of the cutoff parameter on the patient set and the found biomarkers

**Figure 12:**
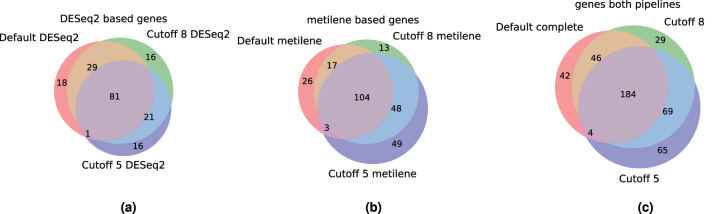
Overlap of identified markers for treatment success without cutoff and with cutoffs of 5 and 8, for DESeq2 results, metilene results and the combination of the two. (a) DESeq2: 182 genes. (b) Metilene: 260 genes. (c) Combined: 439 genes.

The cutoff parameter only changes the assignment of a relatively small number of patients (see Tables 5 and 6). However, it can have a profound effect on the number and *p*-values of the identified markers, as shown in Figure 12.

**Table 5:** Turnover from dead to alive group due to the cutoff parameter for the DESeq2 set. Invocation of 448 patients in total.

DESeq2	No cutoff		Cutoff 8		Cutoff 5	
	Vital status	Count	Vital status	Count	Vital status	Count
	Alive	338	Alive	341	Alive	348
	Dead	110	Dead	107	Dead	100
Turnover:	0		3		3 + 7	
Percentage:	0 %		0.67 %		2.23 %	

**Table 6:** Turnover from dead to alive group due to the cutoff parameter for the metilene set. Invocation of 434 patients in total.

Metilene	No cutoff		Cutoff 8		Cutoff 5	
	Vital status	Count	Vital status	Count	Vital status	Count
	alive	333	alive	336	alive	343
	dead	101	dead	98	dead	91
Turnover:	0		3		3 + 7	
Percentage:	0 %		0.7 %		2.3 %	

## 4 Concluding remarks

TREMSUCS-TCGA is a complete, flexible, self contained and reproducible analysis pipeline for the prediction of treatment success based on the TCGA-database. It drastically simplifies the workflow for a practitioner by automatically acquiring and analyzing the expression and methylation data for patient cohorts based on the user’s specification of only cancer types and treatment regimes to be included. TREMSUCS-TCGA provides a multilevel verification of the survival impact of a potential biomarker estimated with help of the patients survival meta data. It produces a single, comprehensive report of the entire analysis for viewing in a web browser. A showcase example demonstrated that the pipeline identifies biomarkers for treatment success that are biologically plausible.

All features derived in the initial differential analyses that lead to the potential biomarkers are also collected within the final tabular result. Together with the survival analyses significances and the set informations, this table can give valuable ancillary informations for scientists working in cancer research for validating, assisting or questioning their findings. Subsequent analysis like a GSEA (see List of abbreviations) can be deployed manually on the basis of the created gene sets.

The current version of TREMSUCS-TCGA is limited by the availability of treatment data. For example, very few patients in the TCGA-LUSC data set received the analyzed treatment. It is not surprising, therefore that TREMSUCS-TCGA did not identify any markers for TCGA-LUSC. The 18 total cases of TCGA-LUSC in treatment nevertheless contribute to the results of the basket analysis. We provide a table with the aggregation of all TCGA projects and the number of cases available for the given treatment within the supplemental materials, also hosted at the git repository [35].

Furthermore, the creation of basket trials to increase case counts is subject to restrictions. It does not always make sense to combine different types of cancer with different molecular features. Preferably, the cancer types combined should be similar, as is the case for the squamous cell carcinomas used for our example set. Another promising combination would be TCGA-GBM and -LGG (see Table A1) with avastin and temodar treatment, since both cancer types belong to the glioblastomas and this combination would include 401 cases.

After the biomarker candidates are determined with the base plot step (see Figure 1) the validation steps invoke the complement patient set, which are the cases not treated with the chosen drug combination. This might be too inclusive, since it is still possible that some of the complement therapies are, at least molecularly, similar to the chosen ones. A specific selection of the therapies which build the complement set may therefore be desirable.

Since the implementation of TREMSUCS-TCGA is based on Snakemake, it is modular, flexible and extensible and with that the whole approach can be developed further with manageable effort. In future versions, it would be desirable to choose the exact complement set of patients which are not treated with the specified treatment for the validation steps. Using TCGA as the basis for the analysis does exclude many modern treatments not yet (sufficiently) covered in the TCGA. Therefore, an extension of the used database beyond the TCGA-projects would be of interest.

As an outlook, an interface that enables the user to incorporate custom data that are not available in a public repository would be of interest since the results of modern clinical studies are often confidential. TREMSUCS-TCGA is designed to make such an extension easy by virtue of requesting and building all required software and environments with the help of pip, Snakemake and conda. It would require, however, that the user’s proprietary data adheres to stringent standards on data and meta-data formats, or the use of a customized importer.

## A Appendix

**Table A1:** List of projects which are accessible with the DESeq2 and metilene tool.

Project abbreviation	Project name
TCGA-CESC	Cervical squamous cell carcinoma and endocervical adenocarcinoma
TCGA-LUSC	Lung squamous cell carcinoma
TCGA-HNSC	Head and neck squamous cell carcinoma
TCGA-ESCA	Esophageal carcinoma
TCGA-BRCA	Breast invasive carcinoma (squamous cell neoplasms)
TCGA-GBM	Glioblastoma multiforme
TCGA-OV	Ovarian serous cystadenocarcinaoma
TCGA-LUAD	Lung adenocarcinoma
TCGA-UCEC	Uterine corpus endometrial carinoma
TCGA-KIRC	Kindney renal clear cell carcinoma
TCGA-LGG	Brain lower grade glioma
TCGA-THCA	Thyroid carcinoma
TCGA-PRAD	Prostate adenocarcinoma
TCGA-SKCM	Skin cutaneous melanoma
TCGA-COAD	Colon adenocarcinoma
TCGA-STAD	Stomach adenocarcinoma
TCGA-BLCA	Bladder urothelial carcinoma (squamous cell neoplasms)
TCGA-LIHC	Liver hepatocellular carcinoma
TCGA-KIRP	Kidney renal papillary cell carcinoma
TCGA-SARC	Sarcoma
TCGA-ESCA	Esophageal carcinoma (squamous cell neoplasms)
TCGA-PAAD	Pancreatic adenocarcinoma
TCGA-PCPG	Pheochromocytoma and paraganglioma
TCGA-READ	Rectum adenocarcinoma
TCGA-TGCT	Testicular germcelltumors
TCGA-THYM	Thymoma
TCGA-KICH	Kidney chromophobe
TCGA-ACC	Adrenochordical carcinoma
TCGA-MESO	Mesothelioma
TCGA-UVM	Uveal melanoma
TCGA-DLBC	Lymphoid neoplasm diffuse large b-cell lymphoma
TCGA-UCS	Uterine carcinoma
TCGA-CHOL	Cholangiocarcinoma

**Table A2:** Translation of flawed or ambiguous drug names.

Drug name variant found	Harmonised conversion
5-flurouracil, 5-fu, 5 fu, fluorouracil, fluorouracil	5-fluorouracil
Anastrozole	Arimidex
Carboplatinum, paraplatin	Carboplatin
Cetuximab	Erbitux
Cisplatinum, cisplatin-xrt	Cisplatin
Erlotinib	Tarceva
Etoposide	Vepesid
Gemcitabine	Gemzar
Ironotecan	Irinotecan
Palixtaxel, taxol, abraxane	Paclitaxel
Panitumumab	Vectibix
Premetrexed, alimta	Pemetrexed
Taxotere	Docetaxel
Vinorelbin, navelbine	Vinorelbine
Temozolomide	Temodar

## List of abbreviations

AC107373.2: gencode gene: ENSG00000272157
API: application programming interface
BAM: binary alignment map
CPU: central processing unit
DAG: directed acyclic graph
DICOM: digital imaging and communications in medicine
DMR: differentially methylated regions
FDA: US food and drug administration
GDC: genomic data commons data portal
GSEA: Gene set enrichment analysis
ICI: immune checkpoint inhibitor
MLE: maximum likelihood estimate
MMRCS: Mismatch repair cancer syndrome
NEFL: neurofilament triplet L protein
NSCLC: non-small-cell lung cancer
ORR: overall responce rate
PEVO: data solutions based on a basket prospective trial with
RAM: random-access memory
RBP1: retinol binding protein 1
SCC: squamous cell carcinoma
TCGA: the cancer genome atlas
LINC00619: long intergenic non-protein coding RNA 619
lincRNA: long intergenic non-protein coding RNA
lncRNA: long non-coding RNA
ST8SIA6-AS1: ST8SIA6 antisense RNA 1
TREMSUCS-TCGA: TREatMentSuCceSs-TCGA
baseMean: mean of normalized counts for all samples
lfcSE: standard error: condition treated versus untreated
log2FoldChange: log2 fold change (MLE): condition treated versus untreated
padj: BH adjusted *p*-values
*p*value: Wald test *p*-value: condition treated versus untreated
ps: processes
stat: Wald statistic: condition treated versus untreated
